# Supplementary material for: MHCXGraph: a graph-based approach to detecting T-cell receptor cross-reactivity
Source: Brief Bioinform. 2026 Oct 8;27(5):bbag555. doi: 10.1093/bib/bbag555 (PMC13647279; doi:10.1093/bib/bbag555)
Supplement: Supplementary file 1 [file mhcxgraph_bb_supplementary_reviewed_round2_bbag555.pdf]

Supplementary Material

MHCXGraph: A Graph-Based approach to detecting T cell receptor cross-reactivity

Carlos Daniel Marques Santos Simões[1,2], Rocio Lucia Beatriz Riveros Maidana[1], Samuel Chagas de Assis[1,2],
Joao Victor da Silva Guerra[1], Helder Veras Ribeiro-Filho[1,2,*]

[1]Brazilian Biosciences National Laboratory, Brazilian Center for Research in Energy and Materials, Campinas, São Paulo, Brazil
[2]Graduate Program in Pharmaceutical Sciences, Faculty of Pharmaceutical Sciences, University of Campinas, Campinas, São Paulo, Brazil

*Correspondence: helder.ribeiro@lnbio.cnpem.br

1 Characterization of pMHC structural interfaces with TCRs

1.1 Construction of the TCR:pMHC structure dataset for structure analysis

A set of TCR structures bound to pMHC was collected from the TCR3d database [1] on June 23, 2025, without restriction to species. A total of 262 MHC-I and 96 MHC-II PDB entries were retrieved, and only structures with resolution better than 2.5 Å were retained, resulting in 97 MHC-I and 15 MHC-II structures selected for further analysis. PDB ID 9EJI (an MHC-II structure) was excluded due to its unconventional binding mode, which lacks contacts with the peptide. PDB ID 2VLJ was also excluded as it is redundant with PDB ID 1OGA, which has a higher resolution. Among these structures, 14 MHC-I structures and 4 MHC-II structures were derived from mouse, whereas the remaining structures were of human origin.

To ensure compatibility in residue numbering across MHC structures, each collected structure was renumbered according to a IMGT numbering scheme as described in this Supplementary Material.

The bound complexes were analyzed to identify pMHC residues in contact with the TCR, using a 5 Å distance threshold between all atoms. For each PDB, contact residues were determined and then aggregated to generate a list of MHC-I and MHC-II residues observed to interact with the TCR in at least one analyzed structure. In structure analysis, the RSA was computed with the DSSP program (version 4.0.4), using the Wilke scale [2].

1.2 Characterizing pMHC structural interfaces with TCRs to support MHCXGraph analysis

To support MHCXGraph analysis and parameter selection, we first represented and characterized, from a graph perspective, the pMHC (Class I and Class II) interfaces interacting with TCRs in solved 3D complex structures.

From the analysis of 97 MHC class I and 15 MHC class II structures in complex with TCRs from the PDB, we identified 58 distinct MHC positions for MHC-I and 35 for MHC-II that make contact with the TCR in at least one complex, considering a 5 Å distance cutoff (Table S1). Positions 18, 77, and 142 in MHC-I; position 52 in MHC-IIα; and positions 57, 71, and 78 in MHC-IIβ were identified exclusively in mouse complexes and are marked with an asterisk in Table S1. These positions are provided to the user as input residue selectors, representing target graph nodes when constructing surface graphs in the MHCXGraph pipeline. This allows the method to focus on the most important MHC regions involved in interactions with the TCR. Importantly, only a subset of these positions constitutes the interaction interface in any given

pMHC complex: on average, 18 residues for MHC-I and MHC-II. Peptide residues contribute at least 4 in pMHC-I and 6 in pMHC-II.

**MHC**	**Position**	**Frequency**	**Position**	**Frequency**	**Position**	**Frequency**
**MHC-I**	18*	1.03%	72	91.75%	151	75.26%
	19	11.34%	73	81.44%	152	53.61%
	42	1.03%	75	57.73%	153	4.12%
	43	1.03%	76	90.72%	154	85.57%
	44	2.06%	77*	1.03%	155	100.00%
	54	1.03%	79	30.93%	156	9.28%
	55	2.06%	80	18.56%	157	21.65%
	56	1.03%	83	4.12%	158	85.57%
	58	32.99%	84	5.15%	159	50.52%
	59	12.37%	89	1.03%	161	14.43%
	61	8.25%	108	1.03%	162	27.84%
	62	79.38%	109	1.03%	163	79.38%
	63	21.65%	142*	1.03%	165	1.03%
	64	4.12%	143	3.09%	166	46.39%
	65	90.72%	145	3.09%	167	28.87%
	66	87.63%	146	54.64%	169	3.09%
	68	43.30%	147	19.59%	170	14.43%
	69	95.88%	148	1.03%	171	1.03%
	70	20.62%	149	47.42%		
	71	4.12%	150	84.54%		
**MHC-IIα**	37	40.00%	56	100.00%	63	100.00%
	51	6.67%	58	66.67%	65	53.33%
	52*	13.33%	59	100.00%	66	100.00%
	53	93.33%	60	80.00%	67	6.67%
	55	100.00%	62	100.00%	69	6.67%
**MHC-IIβ**	56	73.33%	65	73.33%	72	40.00%
	57*	6.67%	66	93.33%	73	93.33%
	59	13.33%	67	20.00%	74	40.00%
	60	53.33%	68	6.67%	77	80.00%
	61	13.33%	69	66.67%	78*	6.67%
	62	86.67%	70	20.00%	81	6.67%
	63	93.33%	71*	6.67%		

Table S1: Frequency of MHC-I and MHC-II positions observed at the TCR interface. Positions are reported according to the IMGT numbering scheme. Interface positions were defined as MHC residues with at least one atom within 5 Å of the TCR in experimentally solved TCR:pMHC complexes. Frequencies indicate the percentage of analyzed complexes in which each position was observed at the TCR interface. Asterisks indicate positions observed in contact with TCR only in mouse MHC structures.

The average Relative Solvent Accessibility (RSA) of pMHC residues that are in contact with the TCR 5 Å distance), when analyzing the corresponding unbound pMHC structures (i.e., without the TCR), was 0.3 for both MHC-I and MHC-II cases (Supplementary Figure S1A and S1B). The RSA threshold of 0.1 lies at approximately the 85th percentile for both MHC classes, indicating that it is a suitable threshold for selecting nodes to MHCXGraph analysis.

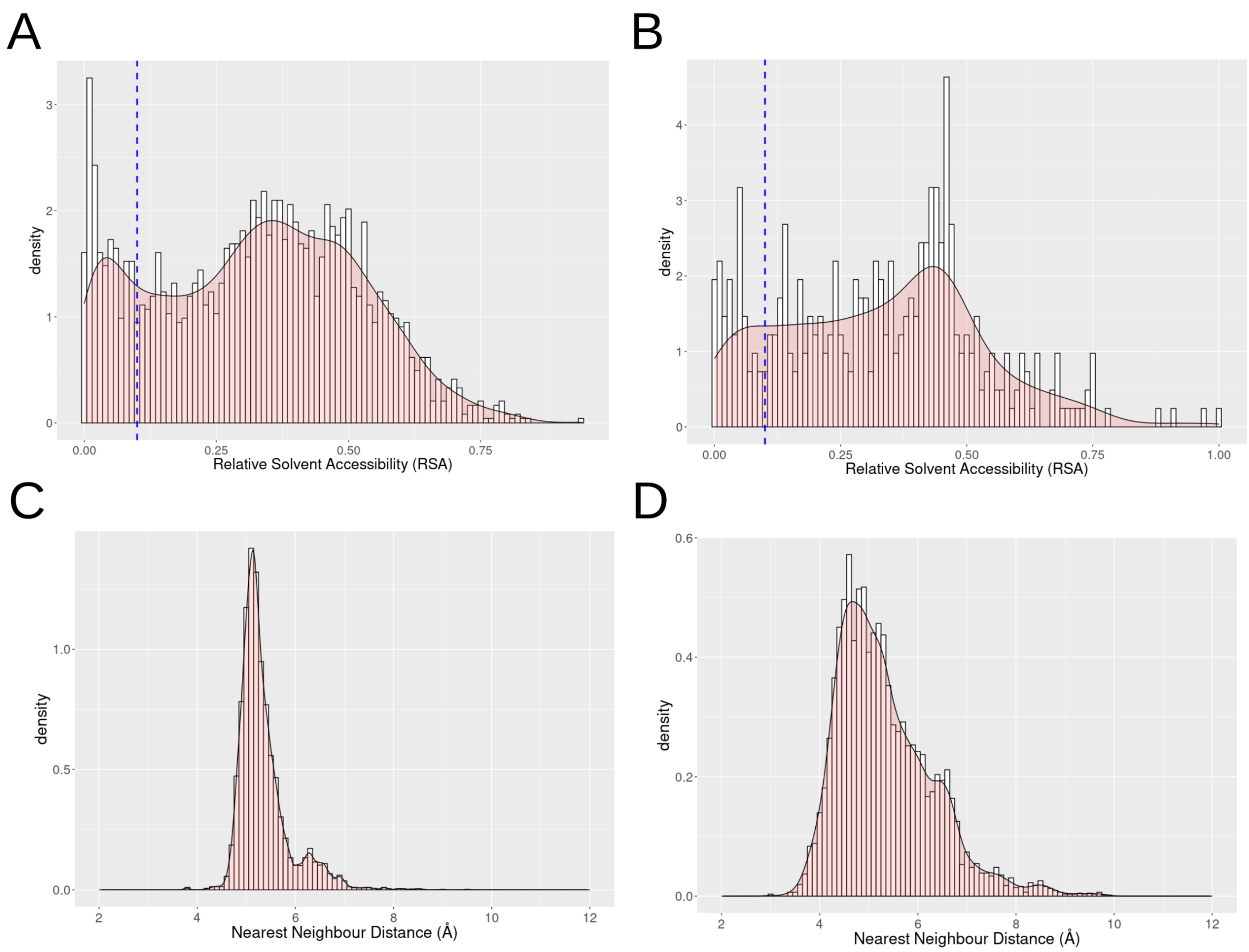

Figure S1: Distribution of Relative Solvent Accessibility (RSA) values for MHC-I (A) and MHC-II (B) residues at the interface with TCRs in TCR:pMHC structures. The vertical blue dashed line indicates an RSA of 0.1, corresponding to the 85th percentile in both MHC classes. Density plots were generated using a binwidth of 0.01. (C) Distribution of the nearest-neighbor distances between the Cα atom of solvent-exposed residues on the upward-facing pMHC surfaces from the set of pMHC-I and pMHC-II structures in TCR:pMHC complexes. Residues from the MHC β-sheet floor were excluded from this analysis: positions 1–50 and 85–136 for MHC-I, and 1-45 (α-chain) and 1-50 (β-chain) for MHC-II. (D) same as (C) but using side chain centroid. In both cases, the direct neighbors of the residues (i-1 and i+1) are not considered. Plots (C) and (D) consider an RSA cutoff of 0.1. Density plots were generated using a binwidth of 0.1.

To propose an appropriate threshold for determining edge formation between nodes in the graphs, we analyzed the distances between exposed residues on the surfaces of pMHC structures and their nearest neighboring residues. We observed an average distance of around 5 Å when using side-chain centroids or Cα atoms (Supplementary Figure S1C and S1D). A distance threshold between 8.5 Å and 10 Å for forming edges between residue nodes would therefore be sufficient to fully cover the analyzed distances when using side-chain centroids or Cα atoms.

2 3D modeling of pMHC structures

For case studies of `MHCXGraph` applications evaluated in this study, pMHC sequences were modeled using the AlphaFold3 server [3] when experimental structures were not available. Only the top-ranked AlphaFold model was used for analysis.

To evaluate computational time and memory usage of `MHCXGraph`, we generated two separate datasets of modeled pMHC structures, each containing 100 entries. The first dataset comprised 100 distinct peptides bound to the same HLA-A*02:01 allele, whereas the second included 100 distinct peptides bound to different MHC-I alleles. All pMHC sequences were obtained from the VDJdb database [4], selecting entries with a confidence score of at least 2 and peptide lengths of 9 or 10 residues. Structures were modeled using ColabFold (v1.5.5) [5], and only the top-ranked models were retained for analysis. Only structures in which the peptides were appropriately bound within the MHC groove in a canonical register were considered.

3 Renumbering of the amino acid sequence in MHC structures

In order to enable users to identify TCR-contacting residues on the MHC and thus restrict the graph search space to only MHC residues observed to interact with TCRs, we provide an accessory program that renumbers the input MHC structure according to the IMGT domain numbering scheme [6]. This program must be executed prior to graph analysis if the user wants to guarantee full compatibility between the list of provided MHC hotspot residues and the MHC structure numbering (Supplementary Material, Table 1).

The renumbering algorithm relies on sequence alignment between the query amino acid sequence of the MHC structure and a list of gapped MHC template sequences (https://www.imgt.org/IMGTrepertoireMH/Proteins/protein/G-DOMAIN/Gdomains.html). The numbering follows the IMGT unique numbering scheme for MHC groove G-DOMAIN and G-LIKE-DOMAIN (https://www.imgt.org/IMGTScientificChart/Numbering/IMGTGsuperfamily.html). MHC-I is treated as two separate domains, $\alpha 1$ and $\alpha 2$, while MHC-II consists of two separate chains, α and β.

The program uses Python scripts built with Biopython and first identifies and extracts MHC-I or MHC-II sequences from the input structures. The extracted sequences are then aligned against all available templates in the ungapped format using global pairwise sequence alignment. A ranking is defined primarily based on the alignment score. In the case of structural gaps in the input structures, residue gaps are inserted into the query sequence representation to account for missing positions, provided that a numbering gap exists in the structure. The pairwise sequence alignment is then projected onto the IMGT numbering positions based on the selected gapped template. Insertions relative to the template are assigned to numbered gap columns when available. In the algorithm, only the MHC sequence in the structure is affected by the renumbering.

4 MHCXGraph execution in chunks

Despite the efficient implementation of the method and extensive use of array operations, increasing the number of protein inputs processed simultaneously can introduce computational challenges in terms of memory usage and execution time on conventional systems, particularly during association triad construction and frame graph generation. To address these limitations, the algorithm processes input proteins in chunks, as specified by the user via the `max_chunks` parameter. Larger chunk sizes enable more proteins to be processed simultaneously but require increased memory resources. Chunks are currently processed sequentially: the first group of protein inputs is processed, followed by subsequent groups. Each chunk yields a set of frame graphs. If, after the initial processing layer, the number of resulting graphs still exceeds the

maximum number of inputs allowed per chunk, an additional layer of chunking is applied. This process is repeated iteratively until completion.

Importantly, a given component within a chunk may generate multiple frame graphs containing redundant association nodes. To prevent the propagation of redundant information to subsequent chunk-processing layers, a union operation is applied to merge all frames into a single graph based on node identifiers, using NetworkX. Although this step may temporarily reintroduce incoherent nodes, this issue is resolved in the final chunk-processing layer, where the union operation is no longer applied and coherence is fully enforced.

Regardless of the value assigned to `max_chunks`, and consequently the number of generated chunks, the resulting coherent frame graphs are unaffected and remain identical. To demonstrate this, we executed `MHCXGraph` in Multiple Mode using the Mel5 case study dataset, comprising nine pMHC structures, with `max_chunks` values of 2, 5, and 9.

As shown in Supplementary Figure S2, both the number of association graph nodes and the number of coherent frame graphs remain unchanged regardless of the number of chunks used during the analysis, illustrating that chunking affects only memory management during computation and does not alter the final results.

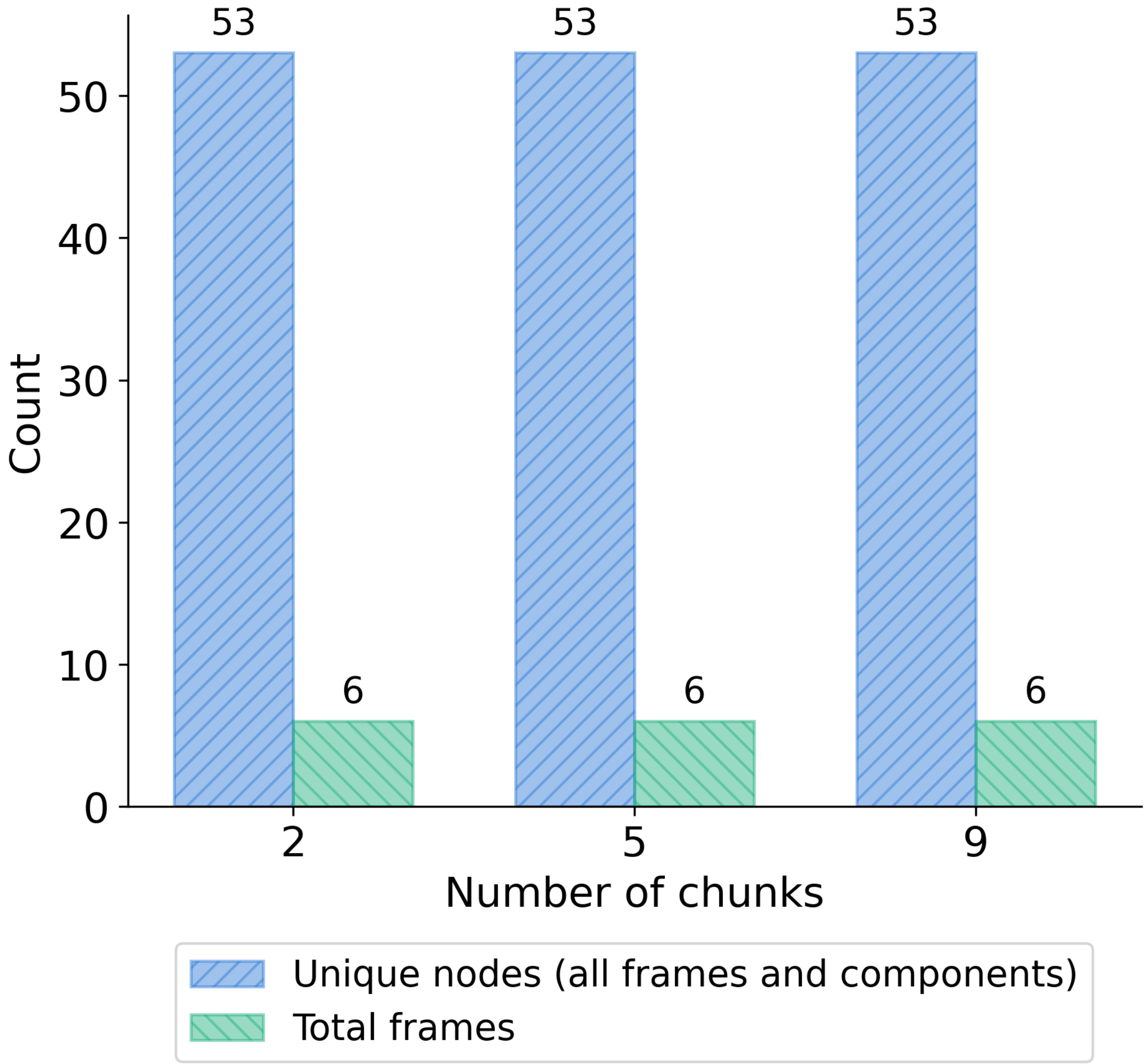

Figure S2: `MHCXGraph` sensitivity analysis of chunk size. `MHCXGraph` was executed in Multiple Mode with all parameters fixed except for `max_chunks`, which was varied from 2 to 5 and 9. The same pMHC structures used in the Mel5 case study were analyzed. The plot summarizes the results obtained for each `max_chunks` value, reporting the number of unique association graph nodes (considering all coherent frames across all connected components) and the total number of generated coherent frame graphs.

5 Determination of chirality of node triads

To compose the descriptors of triads in tokens, a discrete class representing the chirality of the triad is determined. This descriptor assigns a pose-invariant (rigid-body invariant) and mirror-variant chirality label to each triad using local geometry derived from from Cα positions and side-chain direction vectors defined by Cβ coordinates (real Cβ when present, or a virtual Cβ for glycine).

For a triad of nodes (v_i, v_j, v_k) with Cα coordinates $a_i, a_j, a_k \in \mathbb{R}^3$ and Cβ coordinates $b_i, b_j, b_k \in \mathbb{R}^3$, an unit normal vector of the Cα triangle is defined as the normalized cross product of two edges of the Cα triangle $\mathbf{n} = \frac{(\mathbf{a}_j - \mathbf{a}_i)\times(\mathbf{a}_k - \mathbf{a}_i)}{\|(\mathbf{a}_j - \mathbf{a}_i)\times(\mathbf{a}_k - \mathbf{a}_i)\|}$.

By using the Cβ coordinates, for each node $r \in \{v_i, v_j, v_k\}$ an unit side-chain direction is determined as $\mathbf{S}_r = \frac{\mathbf{b}_r - \mathbf{a}_r}{\|\mathbf{b}_r - \mathbf{a}_r\|}$.

Then, for each node, the signal is computed as $\sigma_r = \text{sign}(\mathbf{n} \cdot \mathbf{S}_r)$. The majority signal across the three nodes is identified and when (majority_only = TRUE) (default), only nodes with the majority signal are kept. Exact zeros are mapped to $+1$ for numerical stability.

After majority filtering, let $K \subseteq \{v_i, v_j, v_k\}$ denote the set of residues whose side-chain directions agree with the majority side of the triad plane. The side-chain directions of the retained residues are combined by simple vector summation $\mathbf{v} = \sum_{r \in K} \mathbf{S}_r$.

The averaged side-chain direction is then defined as the unit-normalized vector $\mathbf{s} = \frac{\mathbf{v}}{\|\mathbf{v}\|}$. This normalization ensures that the subsequent chirality score depends only on the direction of the dominant side-chain orientation and is independent of the magnitude of side-chain agreement.

After computing the unit normal vector of the triad plane, n, and the averaged side-chain direction, s, we define a continuous chirality score as their dot product score $= \mathbf{n} \cdot \mathbf{s}$.

Because both vectors are normalized, the score lies in the interval $[-1, 1]$ and corresponds to the cosine of the angle between the triad plane normal and the dominant side-chain direction.

The final discrete chirality label is obtained as $\chi = \text{sign}(\text{score})$ with exact zero values mapped to $+1$ by convention to ensure deterministic behavior.

To be able to perform the calculations when a glycine is presented in the node triad, a virtual Cβ is defined for the glycine residues, using only backbone atom coordinates, following a simple and stereochemically consistent geometric approximation. First, the the Cartesian coordinates of the backbone atoms N, Cα, and C are extracted and denoted as r_n, r_{ca}, r_c, respectively.

Two normalized backbone direction vectors are first computed as $\hat{\mathbf{n}} = \frac{\mathbf{r}_n - \mathbf{r}_{ca}}{\|\mathbf{r}_n - \mathbf{r}_{ca}\|}$ and $\hat{\mathbf{c}} = \frac{\mathbf{r}_c - \mathbf{r}_{ca}}{\|\mathbf{r}_c - \mathbf{r}_{ca}\|}$.

These vectors are combined to define an approximate side-chain direction $\hat{\mathbf{b}} = \frac{\hat{\mathbf{n}} + \hat{\mathbf{c}}}{\|\hat{\mathbf{n}} + \hat{\mathbf{c}}\|}$. The virtual C$\beta$ position is then placed along this direction at a fixed distance from the Cα atom as $\mathbf{r}_{\text{virt}C\beta} = \mathbf{r}_{ca} - d_{ca\text{-}cb}\,\hat{\mathbf{b}}$, where $d_{ca} - d_{cb}$= 1.522 Å corresponds to the canonical Cα – Cβ bond length.

6 MHCXGraph runtime and memory benchmark in Pairwise mode

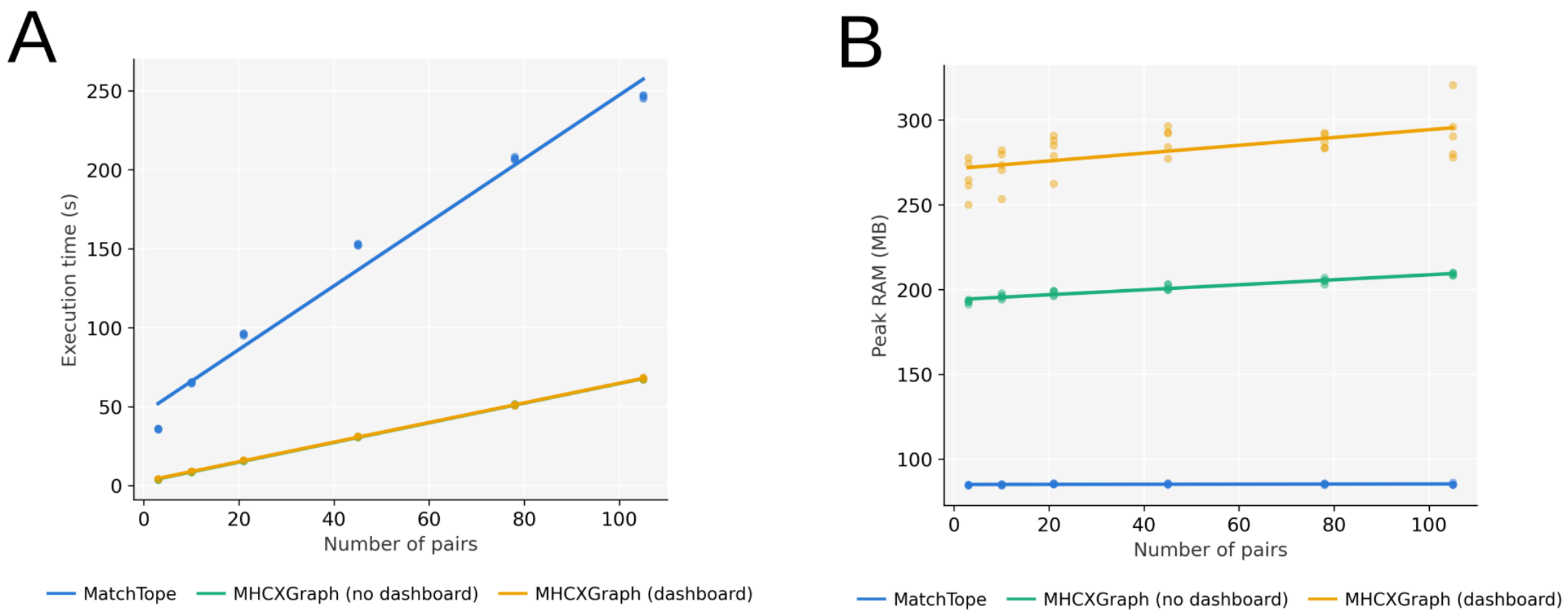

Figure S3: `MHCXGraph` Pairwise Mode runtime and memory usage analysis compared with MatchTope. (A) Runtime analysis of `MHCXGraph` in Pairwise Mode using the Diverse HLA dataset (comprising pMHC structures with different MHC-I alleles and peptides), with and without dashboard generation, and comparison with MatchTope using the same dataset. Runtime is shown as a function of the number of analyzed pMHC pairs. (B) Same analysis as in (A), showing peak RAM memory usage. Linear trend lines are shown for all analyses. For all analyses, `MHCXGraph` was executed using Cα node granularity, an `edge_threshold` of 8.5 Å, and without incorporating RSA into the triad features. The `local_distance_diff_threshold` and `global_distance_diff_threshold` were set to 1 Å and 2 Å, respectively. MatchTope was executed using its default settings from the official repository (https://github.com/Marcus-Mendes/MatchTope).

7 MHCXGraph screening mode for an alphavirus-derived peptide

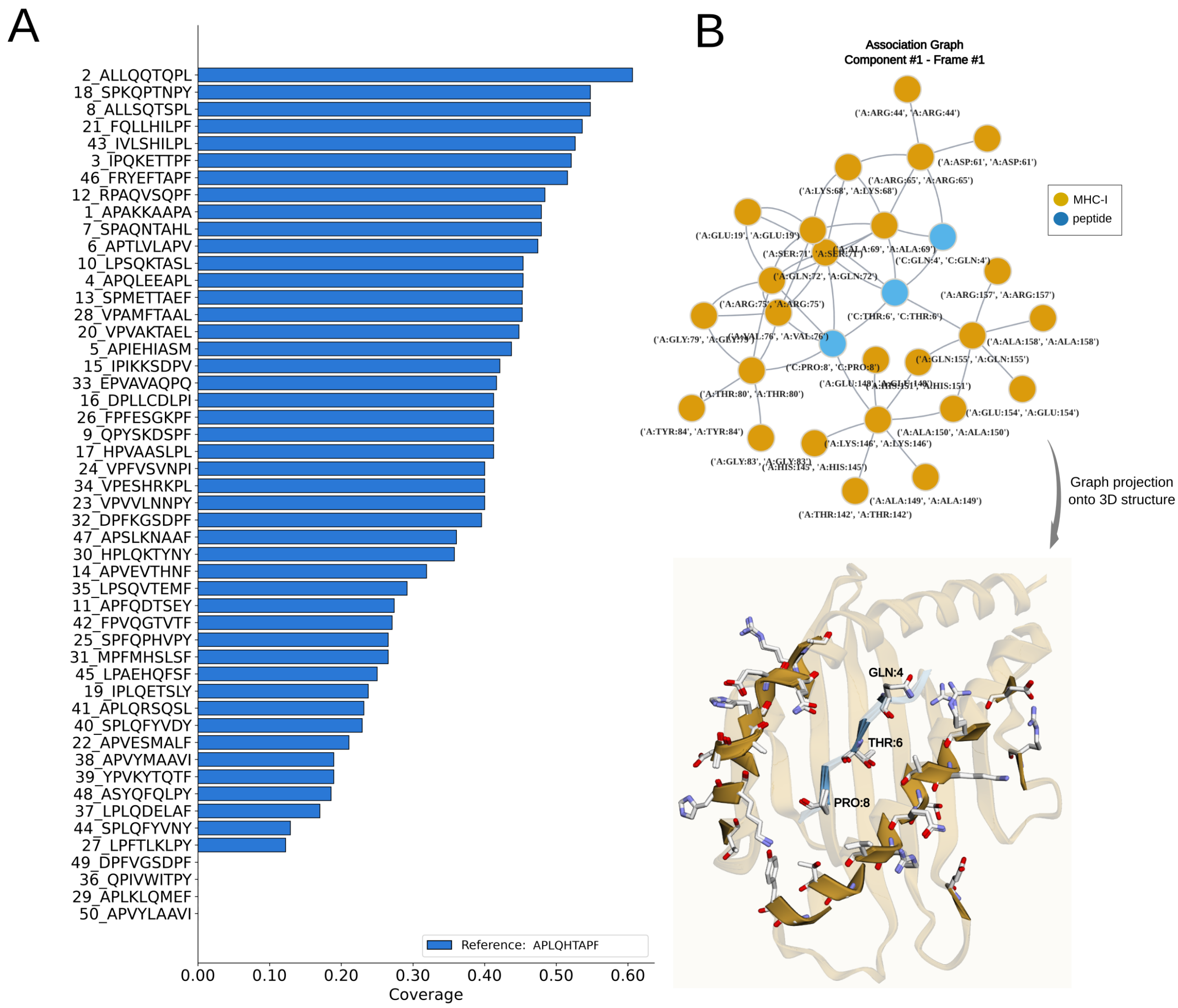

Figure S4: `MHCXGraph` Screening Mode applied to an alphavirus-derived peptide. (A) Ranking of pMHCs (identified by their peptide sequences) according to the coverage-based similarity metric obtained with `MHCXGraph` in Screening Mode, using the alphavirus-derived peptide APLQHTAPF as the reference. The analysis was performed with the `filter_triads_by_chain` parameter enabled to focus the comparison in the peptide chains and their MHC neighboring residues, as all candidate peptides are presented by the same HLA-A*02:01 allele. The candidate peptides originated from an immunopeptidomic screening performed with CrossDome. The first number in each peptide identifier corresponds to its original ranking in CrossDome. CrossDome candidates were obtained using the `cross_background` module (HLA-A*02:01 allele and the default immunopeptidome database), followed by the `cross_compose` module. (B) Largest connected component and its corresponding coherent frame graph for the highest-ranked pMHC identified by `MHCXGraph` (ALLQQTQPL), together with its mapping onto the 3D structure. `MHCXGraph` screening was performed using `ca_only` node granularity, a `local_distance_diff_threshold` of 1 Å, a `global_distance_diff_threshold` of 2 Å, and an `edge_threshold` of 10 Å.

8 Discriminative performance of MHCXGraph and existing methods on a structural pMHC cross-reactivity dataset

To evaluate the discriminative performance of `MHCXGraph` and existing methods in distinguishing cross-reactive from non-cross-reactive cases, we constructed a structural cross-reactivity dataset from the same TCR3d dataset described in Section 1.1 ("Construction of the TCR Structure Dataset for Structural Analysis"). To maximize the number of cross-reactivity cases, we did not filter PDB entries by resolution and included all 262 identified MHC-I structures.

To minimize the effects of unresolved residues and structural gaps, the amino acid sequences associated with all experimental structures were retrieved in FASTA format using their corresponding PDB IDs and annotated as TCRα, TCRβ, epitope, or MHC. To ensure consistent comparisons across TCR sequences, the ANARCI tool [7] was used to extract only the variable domains. Comparisons between TCR sequences were then performed exclusively on the variable domains using exact amino acid sequence identity.

MHC allele assignment was performed to obtain a consistent reference sequence for each MHC molecule represented in the selected PDB entries, enabling comparisons across structures. Assignments were initially performed by searching for exact sequence matches against a reference FASTA database containing MHC allele sequences from multiple species obtained from the IPD-IMGT/HLA Database. When no exact match was identified, BLASTp v2.12.0 was used to align the experimental MHC sequence against the same reference database. Only alignments covering more than 100 residues and containing no gaps were considered. Exact matches were prioritized; otherwise, the alignment with the highest sequence identity was selected when the identity was at least 90%. Amino acid mismatches relative to the assigned reference sequence were recorded to support subsequent manual inspection.

For PDB entries in which the FASTA records represented the peptide and MHC as a single-chain construct, the corresponding sequences were extracted directly from the TCR3d structural annotations. Non-canonical amino acid annotations were used to identify structures containing chemically modified residues. These structures were manually inspected to determine whether the modifications occurred at putative TCR-interacting surfaces. Structures containing non-canonical peptide residues were retained only when the modification was associated with peptide accommodation within the MHC binding groove.

A total of 160 unique TCRs (considering the paired TCRα and TCRβ chains) were identified in the dataset. Among these, 40 TCRs were found in complex with more than one distinct pMHC (differing in peptide sequence and/or MHC allele) and were therefore considered cross-reactive, resulting in a total of 123 experimentally determined cross-reactive pMHC complexes. The final curated dataset comprised 99 unique peptides and 32 distinct MHC-I sequences.

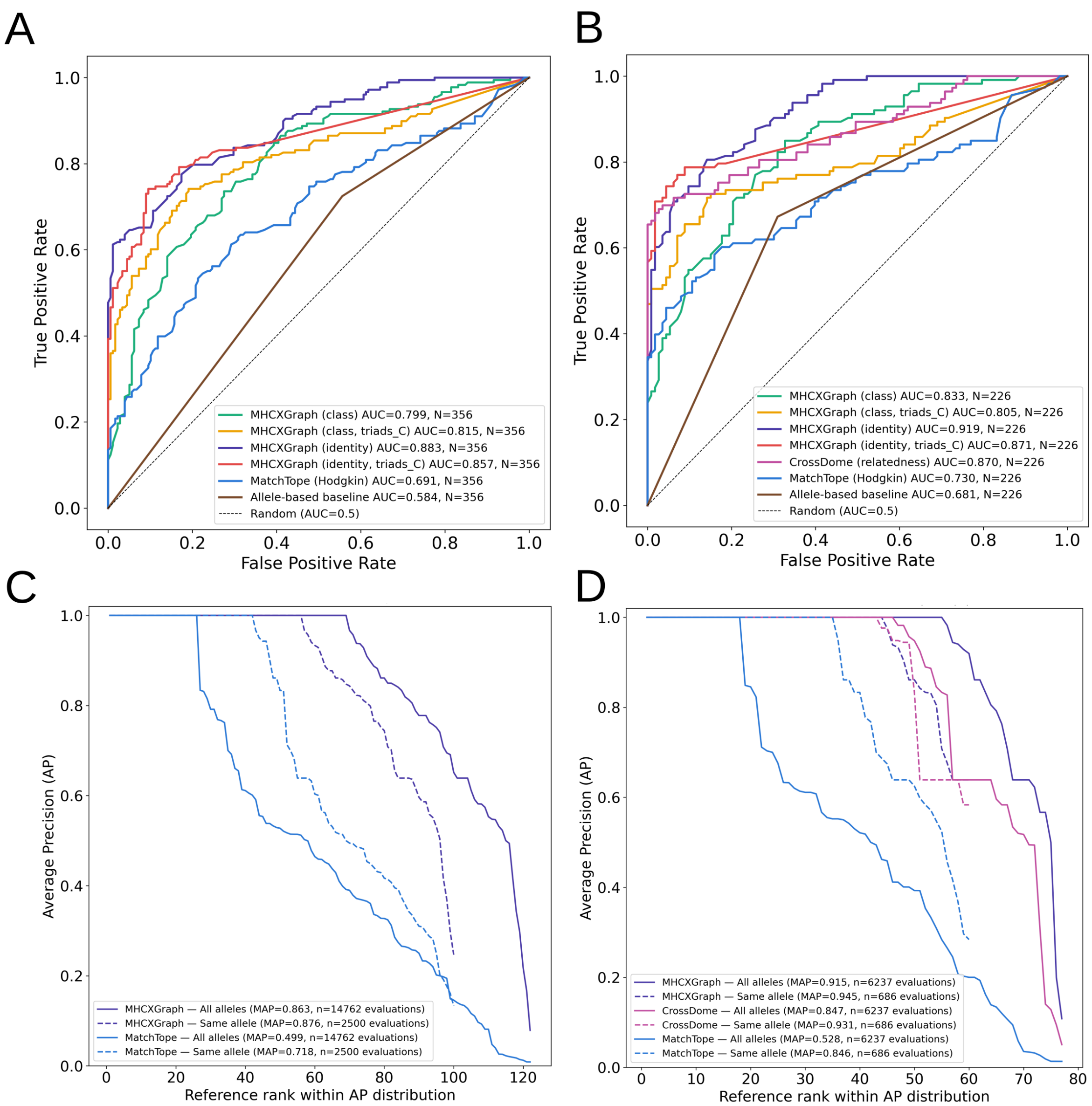

Figure S5: Predictive and ranking performance of `MHCXGraph` and related methods on a structural cross-reactivity dataset. (A) ROC-AUC analysis of `MHCXGraph` using different configurations: amino acid identities (Identity) or amino acid classes (Class), and with the **`filter_triads_by_chain`** parameter enabled to focus the comparison on the peptide and its neighboring MHC residues (Triads_C). For `MHCXGraph`, the coverage-based similarity index was used, whereas the Hodgkin similarity score was used for MatchTope. N represents the number of evaluated pMHC pairs. MatchTope was executed using its default settings from the official repository (https://github.com/Marcus-Mendes/MatchTope). The baseline corresponds to a predictor relying solely on MHC allelic information, i.e., a pair sharing the same MHC allele is predicted as cross-reactive, whereas a pair with different MHC alleles is predicted as non-cross-reactive. (B) Same as (A), but including CrossDome, which is limited to peptides of length 9. All methods were therefore evaluated using the same subset of 9-mer peptides for comparison. (C) Mean Average Precision (MAP) analysis of `MHCXGraph` and MatchTope. For each query pMHC, the Average Precision (AP) was calculated by ranking either all other pMHCs in the dataset (All alleles) or only pMHCs with the same allele as the query (Same allele) according to their similarity score and evaluating the retrieval of cross-reactive pMHCs. MAP corresponds to the mean of the AP values across all query pMHCs. For visualization, AP values are sorted in descending order. (D) Same as (C), but including CrossDome using the subset of 9-mer peptides. N represents the number of all evaluated pMHC pairs across all references.

The curated structural cross-reactivity dataset was then used to evaluate the discriminative and ranking performance of `MHCXGraph` and the other methods.

For the discriminative performance analysis (Supplementary Figure S5), positive (cross-reactive) pairs were generated by considering all pMHC pairs that share at least one TCR, resulting in a total of 178 positive pairs. To generate the negative pairs, pMHC complexes were first organized into groups based on shared TCR relationships: two pMHCs belong to the same group if they share a TCR directly or if they are linked through a chain of intermediate pMHCs that share TCRs. Negative pairs were then defined as pMHC pairs sampled from different groups, ensuring that no TCR relationship exists between the two members of each pair. Sampling was formulated as an integer linear programming optimization problem subject to three constraints: (A) similar individual pMHC frequencies between the positive and negative sets, (B) similar allele-pair frequencies between the positive and negative sets, and (C) a 1:1 ratio of positive to negative samples. This resulted in 178 negative pairs and a final dataset comprising 356 pairs.

Because sequence-based methods such as CrossDome are restricted to peptides of identical length, particularly 9-mers, a second benchmarking strategy was employed. In this case, all methods were evaluated exclusively on the subset of 9-mer peptides to enable direct comparison, following the rules described above, with the dataset reconstructed to maintain the same 1:1 positive-to-negative ratio.

Each positive and negative pair was analyzed with `MHCXGraph` in Pairwise Mode (using different configurations, including peptide triad filtering and amino acid identity or physicochemical classes), and the coverage-based similarity metric was recorded. The same pairs were analyzed with MatchTope, from which the Hodgkin similarity index of the molecular electrostatic potentials was obtained, and with CrossDome using the default mode of the cross_pair_summary module, from which the peptide relatedness score (independent of MHC information) was collected. Because lower CrossDome relatedness scores indicate higher similarity, we inverted the sign of the scores to standardize the performance analyses across all methods. These similarity scores were subsequently used to construct ROC curves and calculate the area under the curve (ROC-AUC), allowing the discriminative performance of the different methods to be compared (Supplementary Figure S5).

As a second benchmark, we evaluated the ability of each method to rank cross-reactive pMHCs according to their similarity scores. Using the same structural dataset, each pMHC was compared against every other pMHC, regardless of whether they belonged to the same cross-reactive group, or against pMHCs with the same MHC allele as the query. Similarity scores were computed using each evaluated method, and the resulting pMHCs were ranked accordingly. For each query pMHC, the positions of the experimentally identified cross-reactive pMHCs in the ranked list were used to calculate the Average Precision (AP). The overall ranking performance of each method was then summarized using the Mean Average Precision (mAP) across all query pMHCs (Supplementary Figure S5).

9 MHCXGraph sensitivity analysis

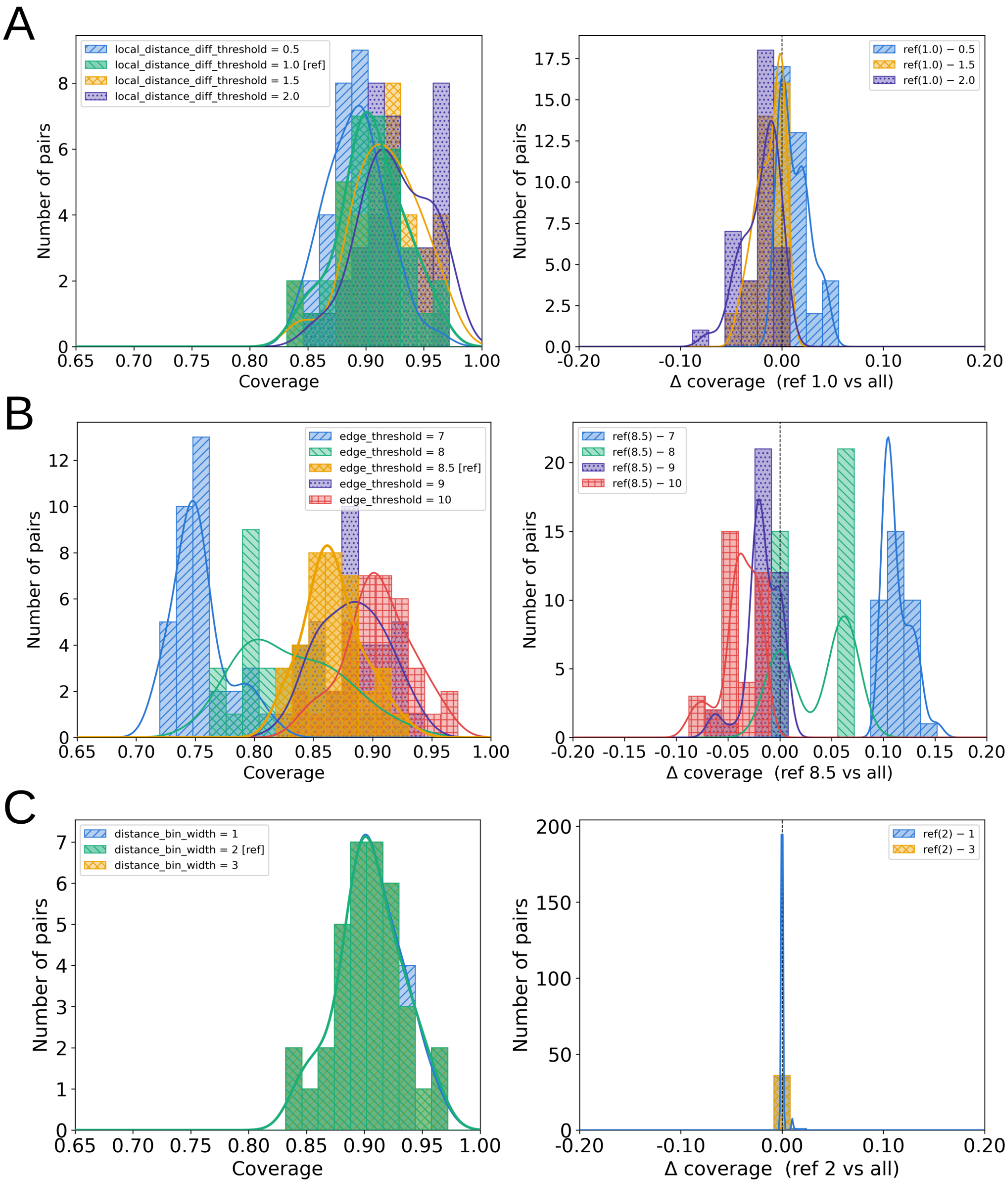

Figure S6: Sensitivity analysis of `MHCXGraph`. `MHCXGraph` was executed in Pairwise Mode using the 36 pMHC pairs from the Mel5 case study. (A) Sensitivity analysis of the `local_distance_diff_threshold` parameter by varying its value from 0.5 to 2 Å while keeping all other parameters fixed. The left panel shows the coverage-based similarity metric, as described in the Materials and Methods section. The right panel shows the difference in coverage relative to the reference value of 1 Å. (B) Same analysis as in (A), varying only the `edge_threshold` parameter from 7 to 10 Å. The right panel shows the difference in coverage relative to the reference value of 8.5 Å. (C) Same analysis as in (A) and (B), varying only the `distance_bin_width` parameter from 1 to 3 Å. The right panel shows the difference in coverage relative to the reference value of 2 Å. All analyses were performed using a `node_granularity` of `ca_only`, an `rsa_filter` of 0.1, a `global_distance_diff_threshold` of 2 Å, and residue selectors corresponding to the MHC interface with TCRs.

10 MHCXGraph analysis with non-canonical amino acids and other molecules

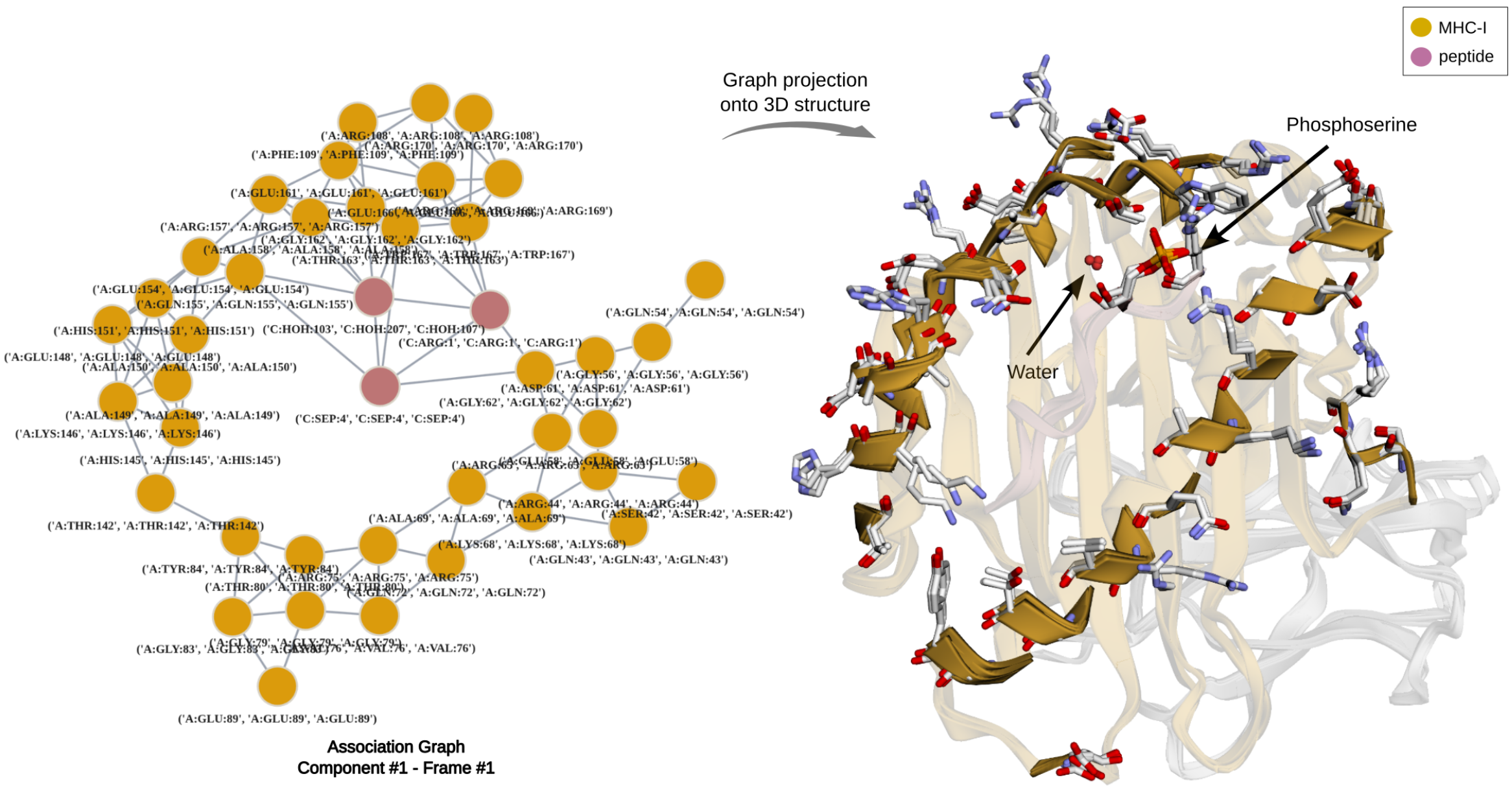

Figure S7: `MHCXGraph` analysis with non-canonical amino acids and other molecules. `MHCXGraph` was executed in Multiple Mode using three PDB structures (4NNX, peptide RQApSLSISV; 4NO2, peptide RQApSIELPSMAV; and 4NO3, peptide RQIpSQDVKL) containing different phosphopeptides presented by the HLA-A*02:01 allele. The analysis was performed with the `include_waters` option enabled, using Cα node granularity, an `edge_threshold` of 10 Å, and `local_distance_diff_threshold` and `global_distance_diff_threshold` values of 1.0 Å and 2.0 Å, respectively. Residue selectors corresponding to the MHC interface with TCRs were used. The left panel shows the largest connected component together with its largest coherent frame graph. The right panel presents the corresponding mapping of this graph onto the 3D structures. The phosphoserine residue and conserved water molecules are indicated by arrows.

11 MHCXGraph parameter description

Table S2: Summary of parameters and flags used for configuring MHCXGraph execution.

Parameter	Description	Type / Accepted Values
run_name	Define the name of the MHCXGraph run	string
run_mode	Define the mode of MHCXGraph execution	string (‘pairwise‘, ‘multiple‘, ‘screening‘)
generate_dashboard	Whether to generate an HTML dashboard file for visualizing the results (default = True).	boolean
dashboard	Activate this flag to automatically open the interactive dashboard for visualizing MHCXGraph results.	boolean
reference_structure	Path to the reference structure when executing the screening mode	string
selectors	List of residues, chain or secondary structures to define the nodes in the protein surface graph	string
filter_triads_by_chain	Specify a chain to restrict triads to those having at least one node from the specified chain. This differs from providing a chain ID in selectors, as it allows focusing on a specific chain and its immediate surroundings (default = None).	string
max_gap_helix	Maximum number of residues between two helices to consider them as a single helix. This is useful when using secondary structure in selectors to treat long helical regions as continuous, even with small breaks (default = 0).	int
output_path	Path to output	string
max_chunks	Maximum number of input structures processed within a single group or batch during execution (default = 5).	int
debug_logs	Activate generation of logs for debugging purposes (default = false).	boolean
debug_tracking	Activate the generation of outputs from intermediate steps. Not recommended when running a large number of input proteins (default = false).	boolean
edge_threshold	Distance cutoff (Å) used to define edges between nodes. Larger values increase graph connectivity, generating more triads and increasing memory usage and computational time (default = 8.5).	float
node_granularity	Atomic representation used to compute 3D coordinates for each residue as nodes.	string (‘all_atoms‘, ‘ca_only‘, ‘backbone‘, ‘sidechain‘)
include_noncanonical_residues	Whether to consider non-canonical residues (e.g., modified amino acids) as nodes in the graph (default = true).	boolean

Parameter	Description	Type / Accepted Values
include_ligands	Whether to consider ligands as nodes in the graph (default = true).	boolean
include_waters	Whether to consider waters as nodes in the graph (default = true).	boolean
distance_bin_width	Width of the distance bins, used to define the number of classes for discretizing distance values. Higher values increase resolution but also the number of token classes (default = 2.0).	int
local_distance_diff_threshold	Maximum allowed difference between distances d1, d2, or d3 of a given triad compared to another triad to allow association (default = 1.0).	float
global_distance_diff_threshold	Maximum allowed distance difference between two non-adjacent node residues from an input protein compared to the corresponding associated nodes from another protein. This threshold is applied during the frame generation step (default = 2.0).	float
close_tolerance	Threshold value that defines the tolerance for distances d1, d2 or d3 to be placed at the center of the bin (default = 0.1).	float
triad_rsa	Whether RSA values are used in the triad token representation. If false, RSA is ignored and all triads share the same RSA class (default = false).	boolean
rsa_filter	Minimum RSA value required for a canonical residue to be considered exposed and included as a node. Zero means no filtering (default = 0.1).	float
asa_filter	Minimum ASA value required for a non-canonical residue, water or ligants, to be considered exposed and included as a node (default = 5).	float
rsa_bins_width	Width of the RSA bins, used to define the number of classes for discretizing RSA values. Higher values increase resolution but also the number of token classes (default = 0.3).	int
close_tolerance_rsa	Threshold value that defines the tolerance for RSA values to be placed at the center of the RSA bin (default = 0.01).	float
rsa_diff_threshold	Maximum allowed difference in RSAs of node residues from a given triad compared to another triad to allow association (default = 0.3).	float
watch_residues	Specify a protein residue to track during algorithm processing. Useful for debugging (default = None)	string
show_std_edges	Whether to display the standard deviation of distances between association nodes in the dashboard (default = False).	boolean

12 Example of Manifest file

```
{
  "settings": {
    "run_name": "test",
    "run_mode": "pairwise",
    "max_chunks": 5,
    "output_path": "/path/to/output",
    "debug_logs": false,
    "debug_tracking": false,
    "edge_threshold": 8.5,
    "node_granularity": "ca_only",
    "include_ligands": true,
    "include_noncanonical_residues": true,
    "include_waters": true,
    "triad_rsa": true,
    "rsa_filter": 0.1,
    "asa_filter": 5,
    "close_tolerance_rsa": 0.01,
    "local_distance_diff_threshold": 1.0,
    "global_distance_diff_threshold": 2.0,
    "distance_bin_width": 2,
    "close_tolerance": 0.1,
    "rsa_bin_width": 0.3,
    "rsa_diff_threshold": 0.3,
    "max_gap_helix": 5.0,
    "generate_dashboard": true

  },
  "inputs": [
    {
      "path": "/path/to/input",
      "enable_tui": false,
      "extensions": [".pdb", ".cif"],
      "selectors": [
        { "name": "MHC1" }
      ]
    }
  ],
  "selectors": {
    "MHC1": {
      "chains": ["C"],
      "structures": {},
      "residues": {
        "A": [18,19,42,43,44,54,55,56,58,59,61,62,63,64,65,66,68,69,70,
        71,72,73,75,76,79,80,83,84,89,108,109,142,143,145,146,147,148,
        149,150,151,152,153,154,155,156,157,158,159,161,162,163,165,166,167,169,170,171]
      }
    },
     "MHC2": {
      "chains": ["C"],
```

```
      "residues": {
        "A": [37,51,52,53,55,56,58,59,60,62,63,65,66,67,69],
        "B": [56,57,59,60,61,62,63,65,66,67,68,69,70,71,72,73,74,77,78,81]
      }
    }
  }
}
```


References

[1] Ragul Gowthaman and Brian G. Pierce. Tcr3d: The t cell receptor structural repertoire database. *Bioinformatics*, 35:5323–5325, 12 2019.

[2] Matthew Z. Tien, Austin G. Meyer, Dariya K. Sydykova, Stephanie J. Spielman, and Claus O. Wilke. Maximum allowed solvent accessibilites of residues in proteins. *PLoS ONE*, 8:e80635, 11 2013.

[3] Josh Abramson, Jonas Adler, Jack Dunger, Richard Evans, Tim Green, Alexander Pritzel, Olaf Ronneberger, Lindsay Willmore, Andrew J. Ballard, Joshua Bambrick, Sebastian W. Bodenstein, David A. Evans, Chia-Chun Hung, Michael O'Neill, David Reiman, Kathryn Tunyasuvunakool, Zachary Wu, Akvilė Žemgulytė, Eirini Arvaniti, Charles Beattie, Ottavia Bertolli, Alex Bridgland, Alexey Cherepanov, Miles Congreve, Alexander I. Cowen-Rivers, Andrew Cowie, Michael Figurnov, Fabian B. Fuchs, Hannah Gladman, Rishub Jain, Yousuf A. Khan, Caroline M. R. Low, Kuba Perlin, Anna Potapenko, Pascal Savy, Sukhdeep Singh, Adrian Stecula, Ashok Thillaisundaram, Catherine Tong, Sergei Yakneen, Ellen D. Zhong, Michal Zielinski, Augustin Žídek, Victor Bapst, Pushmeet Kohli, Max Jaderberg, Demis Hassabis, and John M. Jumper. Accurate structure prediction of biomolecular interactions with alphafold 3. *Nature*, 630:493–500, 6 2024.

[4] Dmitry V. Bagaev, Renske M.A. Vroomans, Jerome Samir, Ulrik Stervbo, Cristina Rius, Garry Dolton, Alexander Greenshields-Watson, Meriem Attaf, Evgeny S. Egorov, Ivan V. Zvyagin, Nina Babel, David K. Cole, Andrew J. Godkin, Andrew K. Sewell, Can Kesmir, Dmitriy M. Chudakov, Fabio Luciani, and Mikhail Shugay. Vdjdb in 2019: database extension, new analysis infrastructure and a t-cell receptor motif compendium. *Nucleic Acids Research*, 48:D1057–D1062, 1 2020.

[5] Milot Mirdita, Konstantin Schütze, Yoshitaka Moriwaki, Lim Heo, Sergey Ovchinnikov, and Martin Steinegger. Colabfold: making protein folding accessible to all. *Nature Methods*, 19:679–682, 6 2022.

[6] Marie-Paule Lefranc, Elodie Duprat, Quentin Kaas, Madeleine Tranne, Aude Thiriot, and Gérard Lefranc. Imgt unique numbering for mhc groove g-domain and mhc superfamily (mhcsf) g-like-domain. *Developmental & Comparative Immunology*, 29:917–938, 1 2005.

[7] James Dunbar and Charlotte M. Deane. Anarci: antigen receptor numbering and receptor classification. *Bioinformatics*, 32:298–300, 1 2016.
